# Spatio-temporal variability of vegetation and its relation to different hydroclimatic factors in Bangladesh

**DOI:** 10.1016/j.heliyon.2023.e18412

**Published:** 2023-07-19

**Authors:** Swadhin Das, Showmitra Kumar Sarkar

**Affiliations:** Department of Urban and Regional Planning, Khulna University of Engineering & Technology (KUET), Khulna, 9203, Bangladesh

**Keywords:** NDVI, EVI, Google Earth Engine, GIS, Bangladesh, Vegetation dynamics

## Abstract

Bangladesh, known for its remarkable ecological diversity, is faced with the pressing challenges of contemporary climate change. It is crucial to understand how vegetation dynamics respond to different climatic factors. Hence, this study aimed to investigate the spatio-temporal variations of vegetation and their interconnectedness with a range of hydroclimatic factors. The majority of the dataset used in this study relies on MODIS satellite imagery. The Normalized Difference Vegetation Index (NDVI), Enhanced Vegetation Index (EVI), precipitation (PPT), evapotranspiration (ET), and land surface temperature (LST) data from the years 2001 to 2020 have been obtained from Google Earth Engine (GEE). In this study, the temporal variations of the NDVI, EVI, PPT, ET, and LST have been investigated. The findings of the Mann-Kendall trend test indicate noticeable trends in both the NDVI and the EVI. Sen's slope value for NDVI and EVI is 0.00424/year and 0.00256/year, respectively. Compared to NDVI, EVI has shown a stronger connection with hydroclimatic factors. In particular, EVI exhibits a better relationship with ET, as indicated by a r^2^ value of 0.37 and a P-value of 6.81 × 10^−^^26^, whereas NDVI exhibits a r^2^ value of 0.17 and a P-value of 2.96 × 10^−^^11^. Furthermore, ET can explain 17% of the fluctuation in NDVI, and no correlation between NDVI and PPT has been found. The results clarify the significant relationship between the EVI and hydroclimatic factors and highlight the efficiency of the EVI for detecting vegetation changes.

## 1 Introduction

Ecosystems on Earth are significantly influenced by climate change, although not all areas are affected equally [1]. Bangladesh is the country most at risk from climate change in the world [2]. Some of the most varied ecosystems on the planet may be found in Bangladesh. The five primary types of ecosystems that exist in Bangladesh include coastal and marine habitats, inland freshwater ecosystems, terrestrial forest ecosystems, highland ecosystems, and man-made ecosystem [3]. Important ecosystem services, including the provision of water, biodiversity, and natural resources, depend on these ecosystems [4]. Monitoring of natural resources, such as vegetation, freshwater, and wetland systems, is necessary for ecosystem protection [5,6]. Ecological degradation is one of the key issues of global environmental change, which greatly restricts the development of human society and affects the evolution of living things [7]. Over the past 10 years, people all over the world have been impacted by more and more environmental disasters [8]. Changes in LULC may be brought on by both climatic and human activities. Massive land use/land cover (LULC) changes have been accelerated in recent decades by rapid urban growth, which has had a considerable negative impact on the local, regional, national, and global environments, as well as their ecosystems [9]. Additionally, due to global warming, a number of human actions have also had a substantial influence on key ecological functions [10].

Vegetation plays a significant role in reflecting and characterizing the interchange of energy, soil ecosystems, the carbon cycle, and regional human activities on the Earth's surface [11] For various purposes, it is crucial to understand how hydroclimatic fluctuations affect local vegetation. Vegetation is the natural link between soil, the atmosphere and water, and plays an important role in maintaining the global ecological balance [12]. It is a primary source of energy and food, seasonal and interannual variations in the vegetation have an impact on the economic and food security of an area [13].

The primary variables influencing the growth of plants are variations in the climate, especially those associated with precipitation and temperature [14]. The carbon, hydrological, and biogeochemical cycles, as well as the global energy budget, are reflected and regulated by plants in soil-water-atmosphere systems [15]. It also protects against erosion and the flow of silt, which helps stabilize the climate [16]. Because of this, vegetation may operate as an excellent indicator for changes in environmental, meteorological, and hydrological elements such as rainfall, surface temperature, evapotranspiration (ET), and soil moisture through the use of various monitoring indices [17]. ET is a crucial part of the hydrologic system because it accounts for moisture lost to the atmosphere by both plants and the land surface [18]. Several studies have linked vegetation's geographical and temporal variation to different hydroclimatic factors. Due to fast climatic change throughout the world, vegetation-climate interactions have received much interest in recent years [16]. Worldwide sources of evapotranspiration include precipitation, temperature, and solar radiation, which interact to affect vegetative greenness. Sudden changes in precipitation variability and temperature cause vegetation patterns to shift, drought conditions to develop, and forest fires to occur, impacting hydrology and geomorphology. Investigating and tracking the relationship between these hydroclimatic factors and vegetation using various proxies will help in understanding long-term changes in vegetation and evaluating the hydrological response [19].

While also being an integral part of the land surface system, vegetation is connected to the air, soil, water, and other environmental factors [20]. To study vegetation dynamics and their interactions with different climatic and hydrological factors in diverse ecosystems, a number of indicators have been used [21]. One of the best ways to observe these long-term relationships between vegetation greenness and trends in a vast region like Bangladesh is with the use of earth observation satellites. These satellite data offer vegetation indices over a larger range of regional and temporal dimensions, which might be useful for tracking environmental change [22].

In recent decades, our understanding of vegetation modeling, water resource management, and hydrological and environmental evaluation has improved thanks to remote sensing-based vegetation indices [23]. Over the years, other datasets such as MeteoSat, MODIS and Landsat series were used for similar purposes at a variety of temporal and spatial scales [24]. Researchers often utilize the MODIS (moderate resolution imaging spectroradiometer). The resolution of images affects the accuracy of remote sensing [25]. In order to investigate both long-term and large-scale processes and obtain an overview of vegetation cover, remote sensing data has become the primary tool [26]. With varied spectral resolutions, vegetation indices combine red and near-infrared spectrum reflectance to precisely measure crop phenology, vegetation categorization, density, seasonal variations, and gross primary production [27].

Vegetation coverage is an important index for the quantitative evaluation of ecosystem vegetation status [28]. Using remote sensing vegetation indices, such as NDVI, different vegetation types may be distinguished clearly [29]. Around the world, vegetation indices like the Enhanced Plant Index (EVI) and the Normalized Difference Vegetation Index (NDVI) are routinely used to monitor changes in vegetation cover [30]. NDVI or comparable indices have been used mostly in studies studying the relationships between hydroclimatic factors and vegetation dynamics [13]. Some studies have evaluated the dynamics of the Normalized Difference Vegetation Index (NDVI) over time and found that the response is not consistent with variations in weather variables like temperature or rainfall [31]. Since seasonal and inter-annual vegetation patterns cannot be predicted merely by relying on one indicator due to their various limitations, it can be deduced from the results of the previous study that both vegetation indices should be employed for better predictions of vegetation pattern and dynamics. Therefore, utilizing both indices would significantly advance environmental management by enabling stakeholders and policymakers to spot early indicators of land degradation or improvement in particular areas [10].

The objective of this study is to inspect the connection among seasonal and inter-annual NDVI and EVI variations, as well as associated meteorological and hydrological factors, in Bangladesh. NDVI, EVI, PPT, ET, and LST data from Google Earth Engine (GEE) for the last 20 years (2001–2020) were analyzed in this study. The study's major findings have the potential to increase knowledge of Bangladesh's ecohydrological characteristics. This is the first study to look into the long-term greenness of whole Bangladesh's vegetation utilizing the NDVI and EVI, as well as their interaction with hydroclimatic parameters.

## 2 Method and data

### 2.1 Description of the study area

**Fig. 1:**
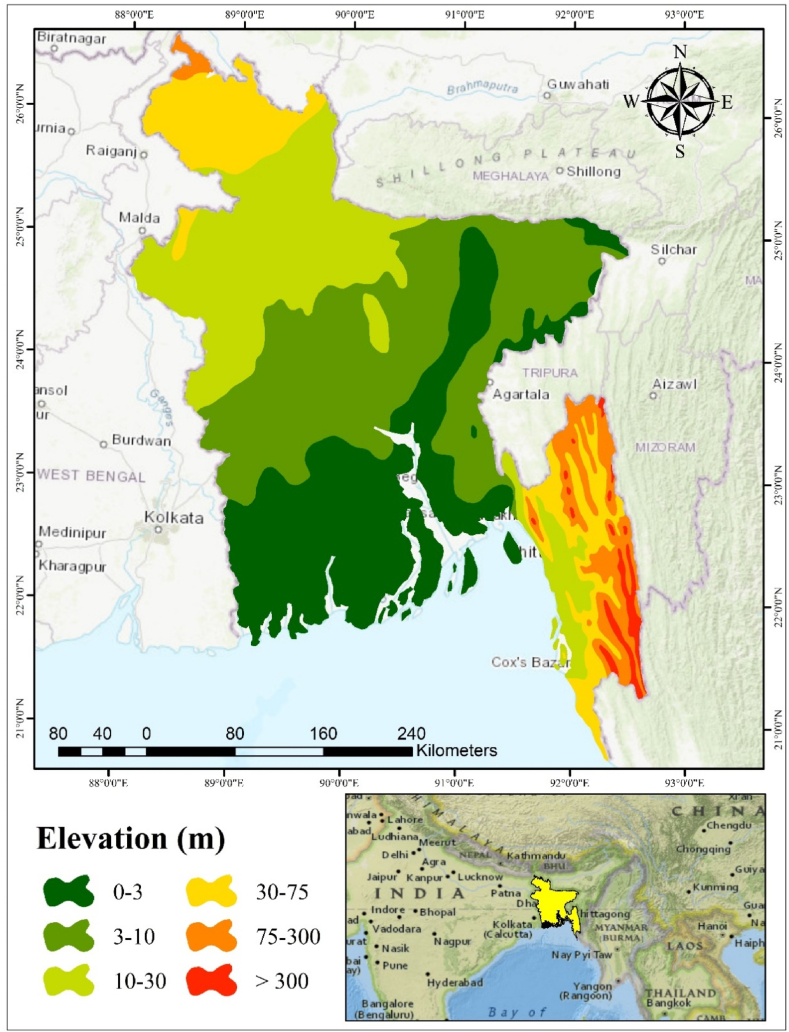
The location of Bangladesh showing elevation.

The research area has been chosen as Bangladesh (Fig. 1). Bangladesh is located between latitudes 20°34′ and 26°38′ north and 88°01′ and 92°41′ east. Bangladesh has a geographical area of 148,460 square kilometers. Bangladesh has the eighth-highest population in the entire world [32]. Its highest and lowest elevations are 1052 m and 0.900 m respectively. It is a riverine country with a coastline of 710 km that is located in a tropical monsoon region. Some of the world's most diversified ecosystems can be found in Bangladesh [3]. High temperatures, a lot of rain, often too much humidity, and very pronounced seasonal fluctuations characterize its climate. It is a very wet country, receiving on average 2200 mm of rainfall per year. Bangladesh's average annual temperature is around 26 °C. Summer is the first month of the Bengali calendar. From the middle of April through the middle of June, the weather is hot and dry, with the odd strong storm. The monsoon season lasts from mid-June to mid-August. Autumn is marked by a steady reduction in temperature and humidity from mid-August to mid-October, with temperatures dropping even further from mid-October to mid-December in late autumn. Winter, which lasts from mid-December to mid-February, is Bangladesh's coldest season. Finally, from mid-February to mid-April, spring arrives [33].

### 2.2 Description of data

All the datasets used in this study were obtained from “Google Earth Engine (GEE)." The monthly precipitation (PPT) data was collected from “GPM IMERG Final Precipitation L3 1 month 0.1° * 0.1° V06’’ from 2001 through 2020 for Bangladesh at an angular size of 0.1° * 0.1° with monthly resolutions. “GPM_3IMERGM” provides a dataset from the years 2000–2021 [34]. That's why this one is suitable for this study.

Information on the land's surface temperature was gathered from “MOD11A2.006 Terra Land Surface Temperature and Emissivity 8-Day Global 1 km” The yearly evapotranspiration (ET) data was gathered from “MOD16A2.006: Terra Net Evapotranspiration 8-Day Global 500 m” which is quite suitable for the Indian subcontinent [10]. Using an 8-day composite dataset, 500-m (m) pixels were produced.

The NDVI and EVI datasets were collected from “MOD13Q1.006 Terra Vegetation Indices 16-Day Global 250 m” at a 16-day temporal resolution and a 250-m spatial resolution. The sources of data applied in this study shown in Table 1.

**Table 1:** Sources of dataset.

Dataset	Source	Accessed On
Precipitation (PPT)	https://developers.google.com/earth-engine/datasets/catalog/NASA_GPM_L3_IMERG_MONTHLY_V06	November 21, 2021
Evapotranspiration (ET)	https://developers.google.com/earth-engine/datasets/catalog/MODIS_006_MOD16A2	November 21, 2021
Land Surface Temperature	https://developers.google.com/earth-engine/datasets/catalog/MODIS_006_MOD11A2	November 21, 2021
NDVI	https://developers.google.com/earth-engine/datasets/catalog/MODIS_006_MOD13Q1	November 22, 2021
EVI	https://developers.google.com/earth-engine/datasets/catalog/MODIS_006_MOD13Q1	November 22, 2021

### 2.3 Analytical methods

**Fig. 2:**
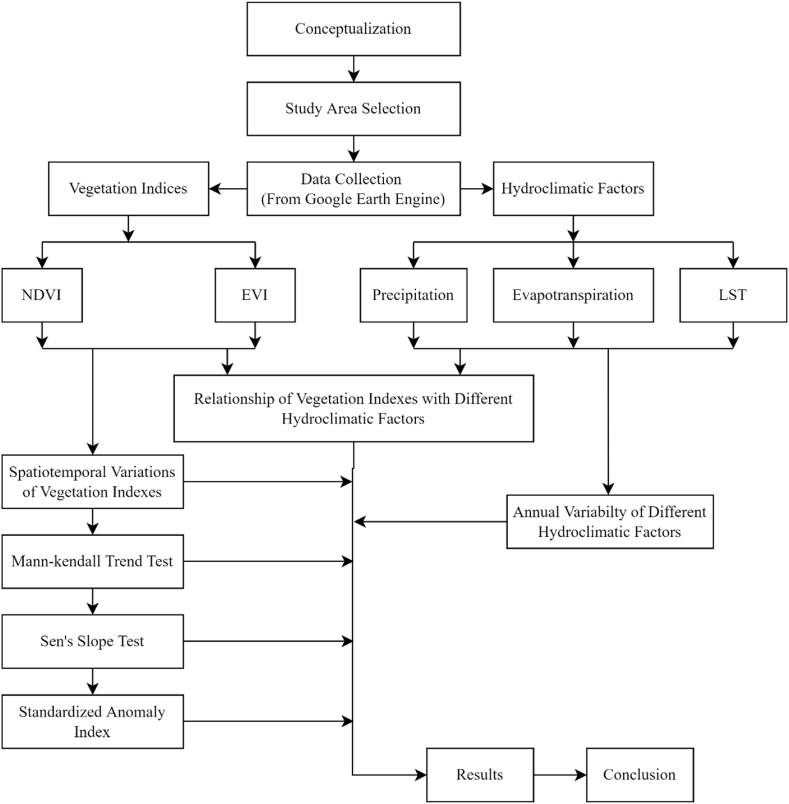
Methodological framework.

Google Earth Engine (GEE) and RStudio took the lead in the majority of the image processing and analysis for this project. The administrative boundary of Bangladesh was used for clipping all the datasets. The values of PPT, ET, T, NDVI, and EVI were obtained for all the pixels in the study area at a monthly interval over 20 years (2001–2020). The methodological framework is shown in Fig. 2.

To examine the variability of NDVI and EVI, the mean value of NDVI and EVI was computed over all pixels. From 2001 until 2020, a mean value was calculated for each month. For analyzing the seasonal variation of NDVI and EVI, the monthly mean from 2001 to 2020 was considered. For analyzing the yearly spatial variation of NDVI and EVI, the yearly mean from 2001 to 2020 was considered and represented in a map made in ArcGIS. The mean spatial variation was determined in order to estimate the coefficient of variation (CV) for the vegetation indices and hydroclimatic parameters. Linear regression was used to find the relationship between NDVI and EVI and rainfall and evapotranspiration. For the trend analysis of NDVI and EVI, two tests were conducted. Both the “Mann-Kendall Trend Test” and “Sen's Slope Calculation” were conducted in RStudio.

## 3 Results

### 3.1 Annual variability of different hydroclimatic factors

**Fig. 3:**
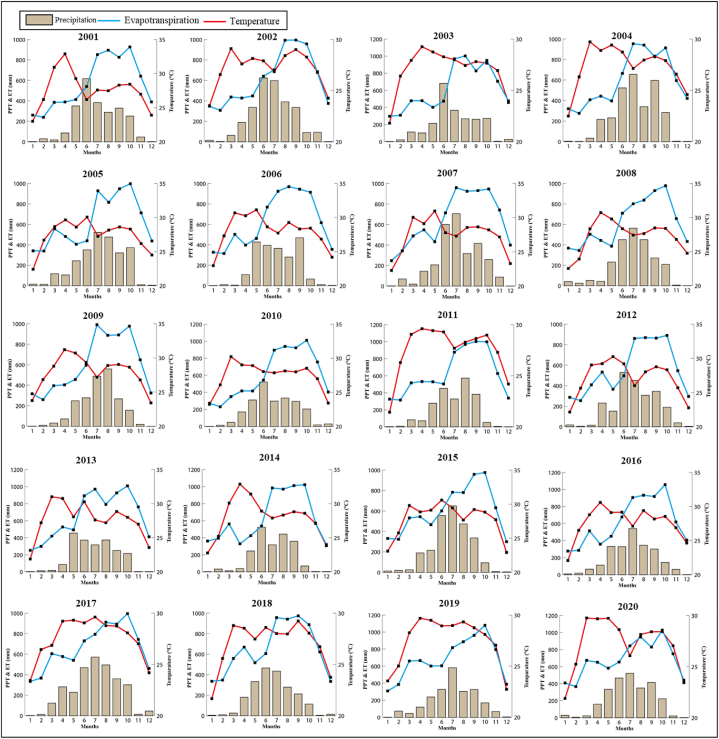
Inter-annual fluctuation of temperature in degrees Celsius, precipitation, and evapotranspiration (ET) in millimeters in Bangladesh during 2001–2020.

Fig. 3 denotes monthly precipitation (mm), evapotranspiration (mm), and mean temperature (°C) for the twenty years (2001–2020) and their inter-annual variability. These graphs are developed from the mean monthly values of the entire Bangladesh. The value of the monthly precipitation for the past 20 years is shown in the bar plots in Fig. 3. These bar graphs demonstrate a consistent trend throughout the course of the 20 years, but the amplitude fluctuates. The monsoon season, which lasts from May to October, is when most of the rain falls. The wettest and driest years of these 20 years were 2017 and 2014, respectively. The red linear graphs represent the mean monthly temperature over 20 years and its temporal pattern for Bangladesh. The mean monthly temperature shows almost the same pattern over the past 20 years. The peak happens between March and May, which is in the summer season. The blue linear graphs represent the total monthly evapotranspiration over 20 years and its temporal pattern for Bangladesh. Like temperature, ET also shows almost the same pattern. The peak happens between June and October. Most of the time, the linear graph of evapotranspiration exceeds the bar plots of precipitation for a specific month.

### 3.2 Temporal variations of vegetation indices

#### 3.2.1 Inter-annual variations of NDVI

**Fig. 4:**
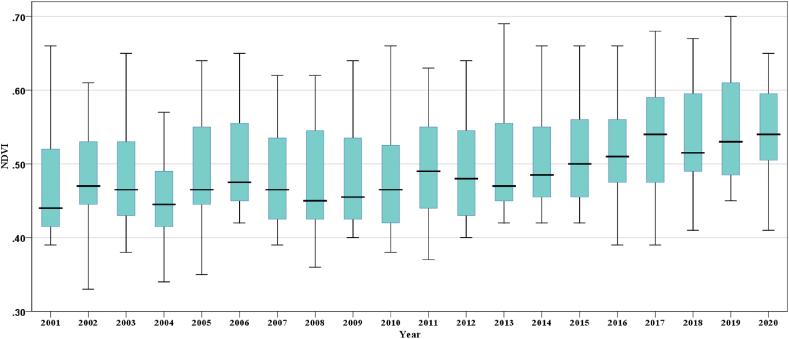
Boxplot showing the NDVI's inter-annual fluctuation in Bangladesh during 2001–2020.

In Fig. 4, the inter-annual variability of NDVI for the year 2001–2020 has been shown in boxplots. The boxplot is developed from NDVI data of all months from January to December ranging from 2001 to 2020 and represents the mean yearly values of NDVI. It shows that NDVI doesn't vary drastically between the periods. Across the years 2001–2020, the 25th percentile of NDVI values started at 0.417 and the 75th percentile of NDVI values ended at 0.612. The minimum value of NDVI is 0.33, which was recorded in June of the year 2002, and the maximum one is 0.70, which was recorded in October of the year 2019. The NDVI varied more and less in 2005 and 2009, respectively. It was discovered that NDVI values were consistently greater than 0.41, indicating that Bangladesh had an adequate amount of vegetation over the time period.

**Fig. 5:**
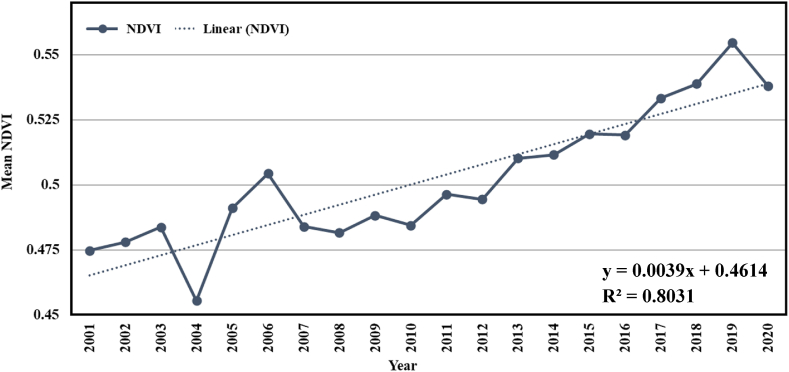
Inter-annual variability of mean NDVI in Bangladesh during 2001–2020.

In Fig. 5, the trend of NDVI has been shown for the years 2001–2020 in Bangladesh. To identify the temporal variation, a linear regression model was developed. The mean NDVI value fluctuates from 0.46 to 0.55. It shows that there was a huge downgrading trend in the mean NDVI between 2004 and 2007. In 2004, there was a devastating flood. The widest extent of the flood was about 35,000 km^2^, which is almost 24% of Bangladesh [35]. In total 36 million people (about 25% of the population) and 39 out of 64 districts were affected [36]. Another flood in Bangladesh in 2007 damaged more than two million hectares of crops and vegetation plots, affecting 252 villages in 40 districts [37]. As the value of water bodies in NDVI is negative, the average value of NDVI fell drastically in 2004 and 2007. But since 2007, the NDVI has been on an upward trend. From 2008 to 2020, the increasing rate of mean NDVI is 0.004/year. So, there is a greening trend within this period. The greater inter-annual fluctuation was greater from 2003 to 2007. The trendline of the mean NDVI shows a growing trend over the years. It indicates that the vegetation's greenness has been increasing since 2001 in Bangladesh. The standard deviation and mean of the annual NDVI were 0.49 and 0.50, respectively.

#### 3.2.2 Inter-annual variations of EVI

**Fig. 6:**
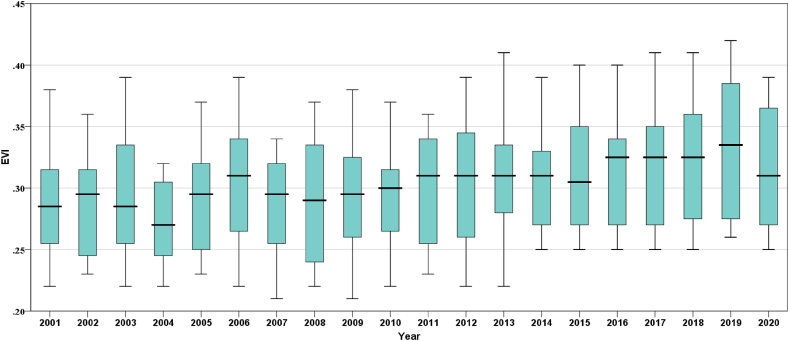
Boxplot for the inter-annual variability of EVI in Bangladesh during 2001–2020.

The inter-annual variability of the EVI for the years 2001–2020 is depicted in boxplots in Fig. 6. The boxplot is developed from EVI data for all months from January to December, ranging from 2001 to 2020 for Bangladesh, and represents the mean yearly values of EVI. It shows that EVI doesn't vary drastically between periods. Across the years 2001–2020, the 25th percentile of EVI values started at 0.24 and the 75th percentile of EVI values ended at 0.38. The range of EVI is not greater than the range of NDVI. The minimum value of EVI is 0.21, which was recorded in January of the years 2007 and 2009, and the maximum value is 0.43, which was recorded in October of the year 2019. The EVI varied more and less in 2005 and 2009, respectively. It was detected that EVI values were always higher than 0.23, which indicated that Bangladesh had enough vegetation throughout the period.

**Fig. 7:**
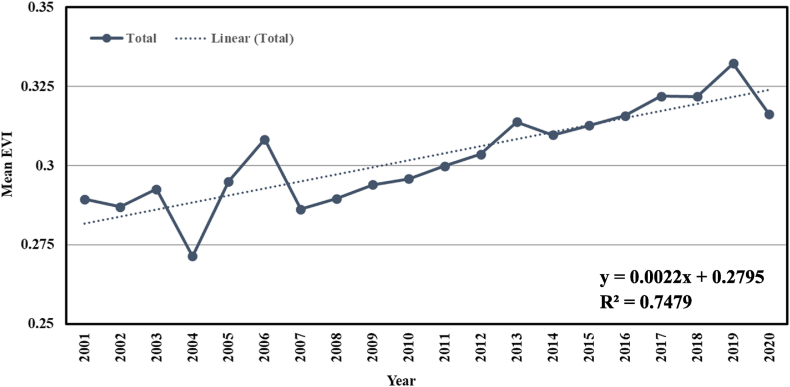
Inter-annual variability of mean EVI in Bangladesh during 2001–2020.

In Fig. 7, the trend of EVI has been shown for the years 2001–2020. To identify the temporal variation, a linear regression model was developed. The mean EVI value fluctuates from 0.27 to 0.33. Like NDVI, there was a huge downgrading trend in mean EVI in 2004 and 2007 because of the floods. But since 2007, there has been an increasing trend in EVI. The growth rate of mean EVI is 0.0025/year, which is less than the growth rate of NDVI. The greater inter-annual fluctuation was greater from 2003 to 2007. The trendline of the mean EVI shows a growing trend over the years. The standard deviation and mean of the annual EVI were 0.050 and 0.29, respectively.

#### 3.2.3 Seasonal variations of NDVI

**Fig. 8:**
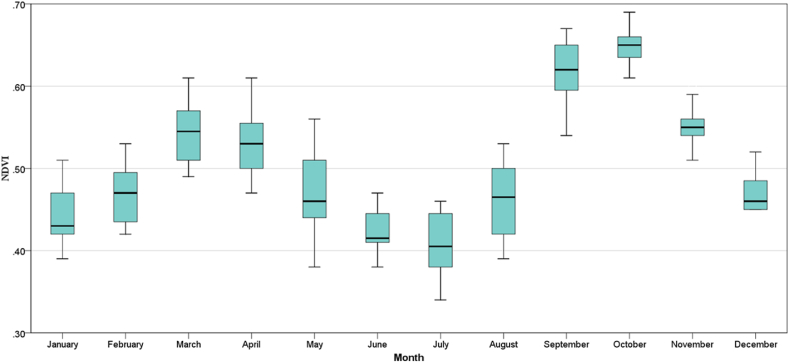
NDVI's average monthly temporal fluctuations in Bangladesh during 2001–2020.

In Fig. 8, the monthly temporal variation of NDVI for the years 2001–2020 has been shown in boxplots. The boxplot is developed from NDVI data ranging from 2001 to 2020 and represents the mean monthly values of NDVI. It shows that NDVI varies significantly between months. The boxplot shows that the NDVI has its highest value in October and its lowest value in July. It also shows that the NDVI value starts to increase in July and reaches its peak in October. After that, in the winter, the value of NDVI started to decrease gradually until spring came. It increased again from spring until summer. After that, there is a decreasing trend until July.

#### 3.2.4 Seasonal variations of EVI

**Fig. 9:**
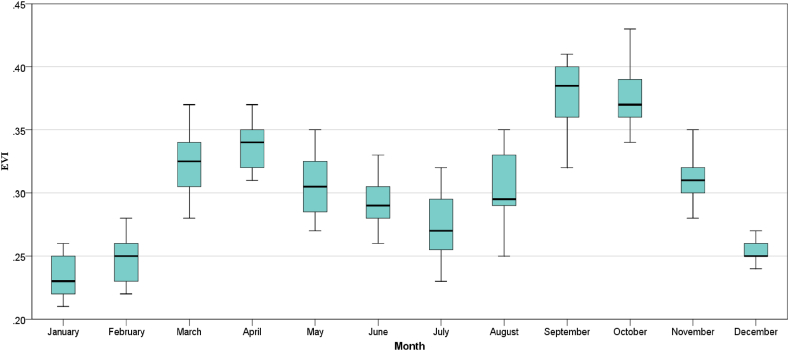
EVI's average monthly temporal fluctuations in Bangladesh during 2001–2020.

In Fig. 9, the monthly temporal variation of EVI for the years 2001–2020 has been shown in boxplots. The boxplot was developed from EVI data ranging from 2001 to 2020 and represents the mean monthly values of EVI. It shows that EVI varies significantly between the months. The box plot shows that the EVI has its highest value in September and its lowest value in January. It also shows that the EVI value starts to increase in July and reaches its peak in September. After that, in the winter, the value of EVI started to decrease gradually until spring came. It increased again from spring until summer. After that, there is a decreasing trend until July.

### 3.3 Spatial variations of vegetation indices

#### 3.3.1 Inter-annual variations of NDVI

For the years 2001 through 2020, Fig. 10 provides geographic maps of long-term mean monthly NDVI. The NDVI shows a greater variance between 2003 and 2007 than the rest of the years. The average NDVI value varies between 0.46 and 0.55. The year 2004 has the brownest pixels across Bangladesh out of all the years. Which means, this year's NDVI values are lower. The tendency is reversed in 2019, with higher NDVI readings across much of the basin.

**Fig. 10:**
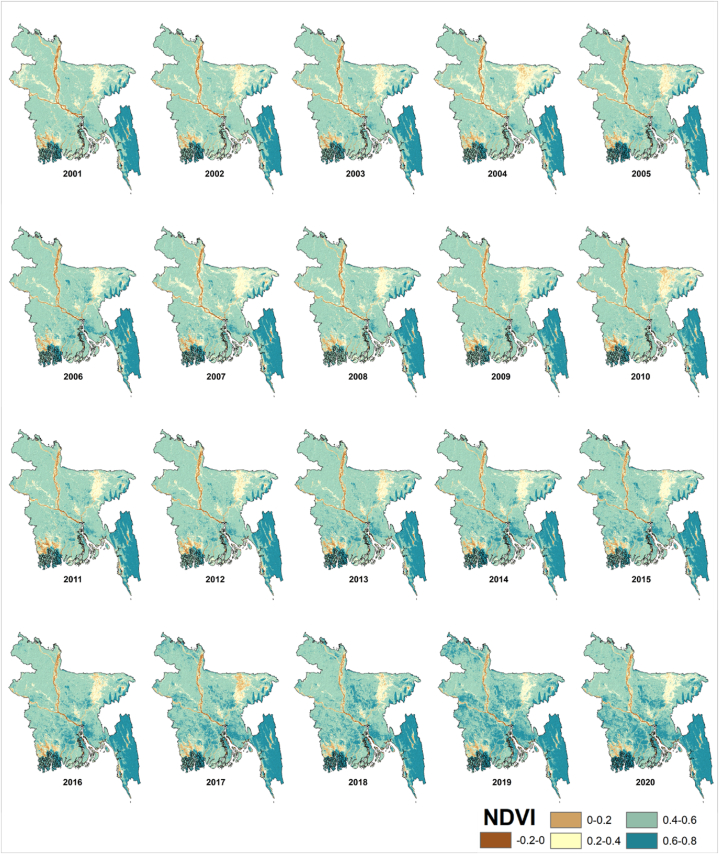
Mean yearly spatial variations of NDVI in Bangladesh during 2001–2020.

#### 3.3.2 Inter-annual variations of EVI

**Fig. 11:**
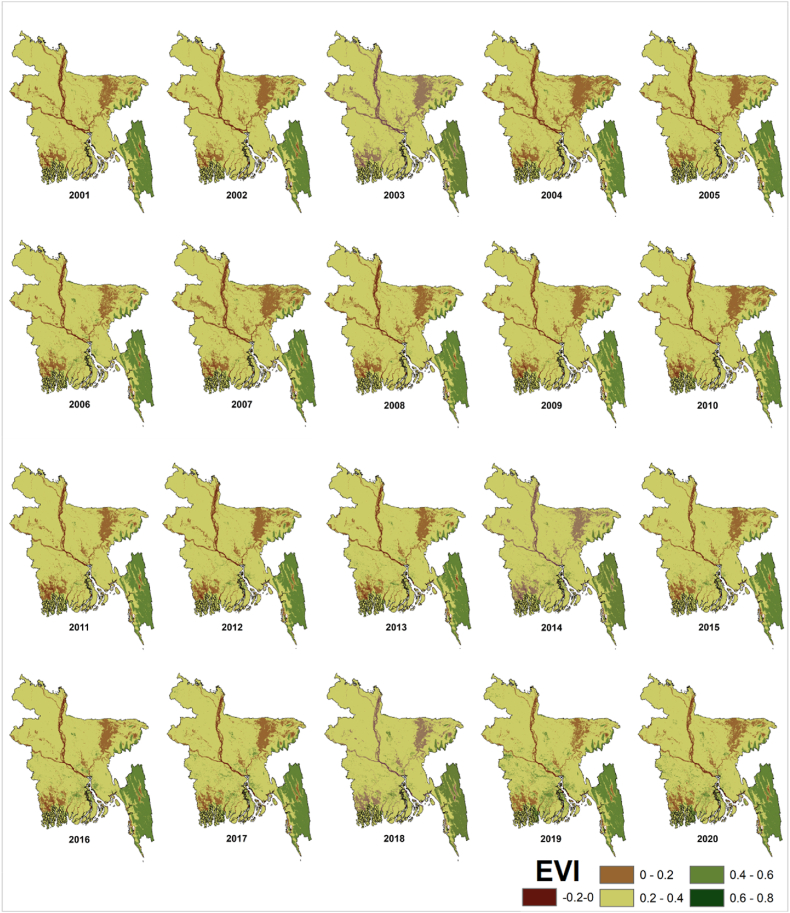
Mean yearly spatial variations of EVI in Bangladesh during 2001–2020.

Similar trends are seen in the EVI's mean yearly spatial analysis in Fig. 11. The average EVI value varies between 0.27 and 0.33. Compared to the mean NDVI, the mean EVI has a narrower range.

### 3.4 Relationship of vegetation indices with different hydroclimatic factors

#### 3.4.1 Average temporal coefficient of variation of several vegetation indices

**Fig. 12:**
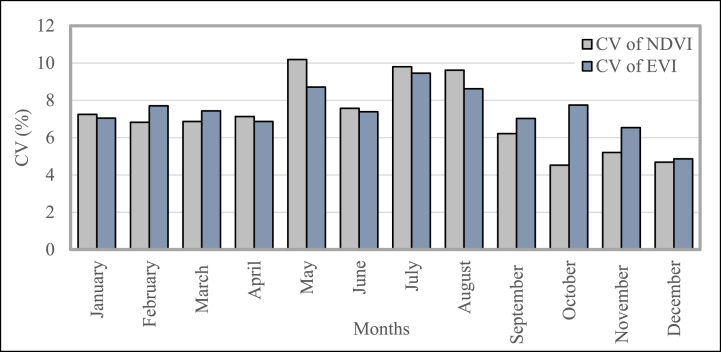
NDVI and EVI mean temporal coefficients of variation (CV in percent) in Bangladesh during 2001–2020.

Fig. 12 represents the coefficient of variation (CV) of dissimilar vegetation indexes measured in this study. It shows the monthly values of CV for NDVI and EVI for the past twenty years. These vegetation indexes show quite the same pattern during all seasons. The variation of NDVI hasn't fluctuated much from January to April. Though the variation increases in May, it decreases in the next month. After that, it increased again in July and started to decrease again until December. EVI shows a similar pattern to NDVI. The higher variation of NDVI and EVI occurred from May to December.

#### 3.4.2 Average temporal coefficient of variation of several hydroclimatic factors

**Fig. 13:**
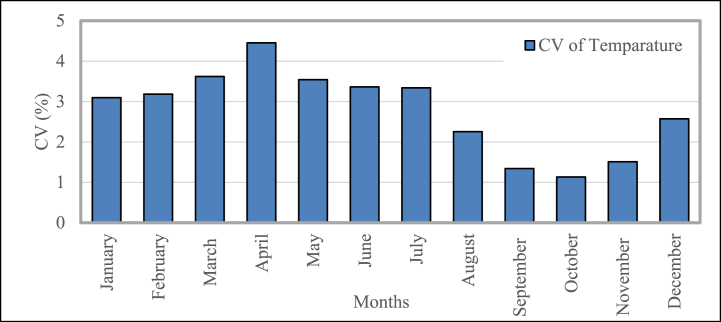
Temperature mean temporal coefficient of variation in Bangladesh during 2001–2020.

The coefficient of variation (CV) of the temperature recorded for this investigation is shown in Fig. 13. It displays the temperature (T) readings for each month over a period of twenty years. The temperature's coefficient of variation climbs from 3% in January to its greatest value of 4.45% in April, then falls to the lowest CV of 1.13% (October). The variation of the temperature from January to July is lower than the variation in the rest of the year.

**Fig. 14:**
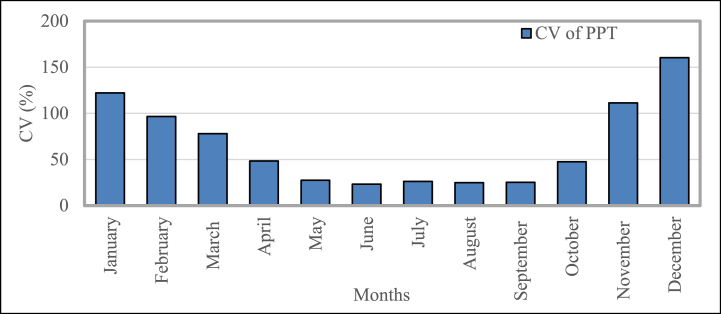
Mean temporal coefficient of variation for precipitation in Bangladesh during 2001–2020.

Fig. 14 represents the coefficient of variation (CV) of rainfall measured in this research. It displays the precipitation (PPT) readings for the past 20 years on a monthly basis. In the CV, the maximum variation is shown by rainfall. The CV of PPT decreases from ∼122% (January), accomplishment its lowest rate ∼23.27% (June), and then rises to its highest value ∼160.31% (December). The variation of the temperature from May to September is lower than the variation in the rest of the year. Because the data points are very distant from the mean, the value of CV for January, November, and December is greater than 100%.

**Fig. 15:**
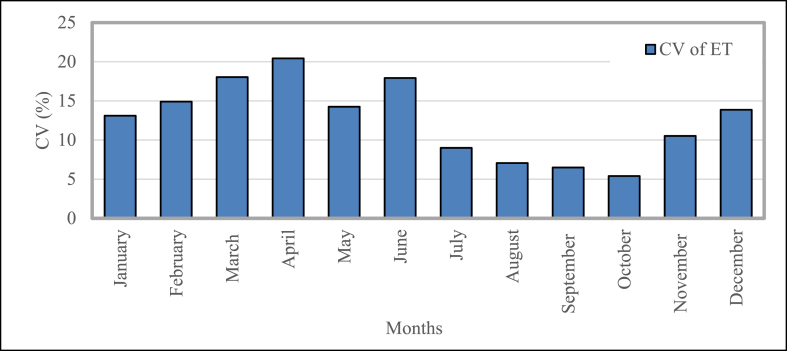
Mean temporal coefficient of variation for evapotranspiration in Bangladesh during 2001–2020.

Fig. 15 represents the coefficient of variation (CV) of evapotranspiration measured in this study. It shows the monthly values of evapotranspiration (ET) for the twenty years. The CV of ET rises from ∼13.11% (January), attainment its peak rate of ∼20.42% (April), and decreases to its lowest value of ∼5.40% (October). The variation of the temperature from April to July is higher than the variation in the rest of the year.

#### 3.4.3 Relationship between mean temporal NDVI and different hydroclimatic factors

**Fig. 16:**
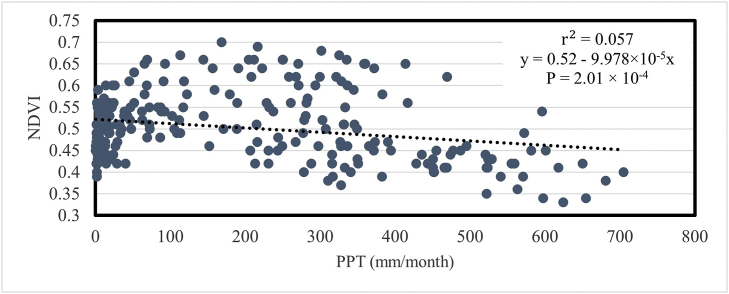
Relationship between mean temporal NDVI and PPT in Bangladesh during 2001–2020.

Fig. 16 represents the relationship of NDVI with monthly precipitation (PPT). The NDVI is not significantly correlated with PPT. In this case, $r^{2}$ = 0.057 and P = 2.01 × 10^−4^.

**Fig. 17:**
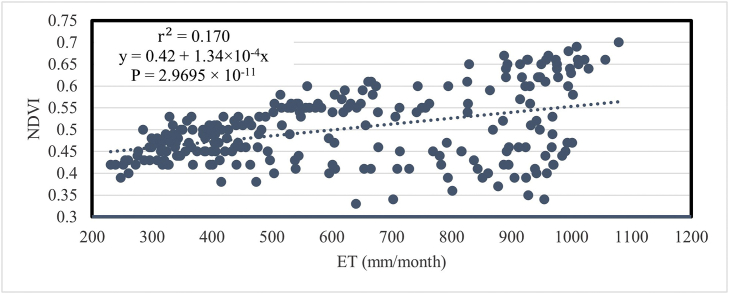
Relationship between mean temporal NDVI and ET in Bangladesh during 2001–2020.

Fig. 17 represents the relationship of NDVI with monthly evapotranspiration (ET). In this case, the value of $r^{2}$ is 0.17 and the value of P is 2.9695 × 10^−11^. That means ET is not explaining much in the variation of NDVI. It can only explain 17% of the variation in NDVI.

#### 3.4.4 Relationship between mean temporal EVI and different hydroclimatic factors

**Fig. 18:**
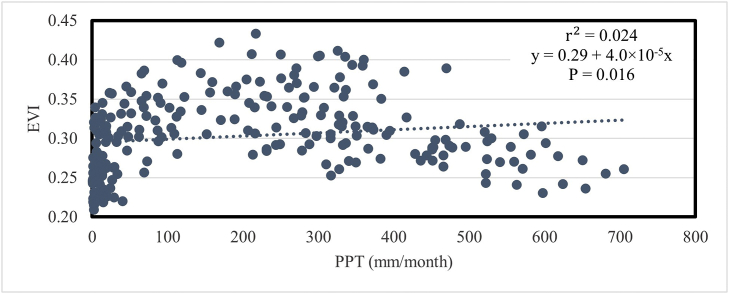
Relationship between mean temporal EVI and precipitation in Bangladesh during 2001–2020.

The link between EVI and monthly precipitation is seen in Fig. 18. The EVI and PPT do not significantly correlate. The value of r^2^ in this instance is merely 0.024, and the P value is 0.016.

**Fig. 19:**
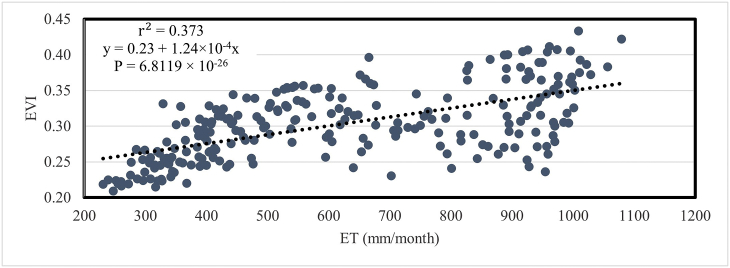
Relationship between mean temporal EVI and ET in Bangladesh during 2001–2020.

Fig. 19 represents the relationship of EVI with monthly evapotranspiration (ET). In this case, the value of $r^{2}$ is 0.373 and P = 6.8119 × 10^−26^. That means EVI displayed reasonable performance. It means a full 37% of the variation of EVI is completely explained by ET.

### 3.5 Trend analysis of vegetation indices

#### 3.5.1 Mann-Kendall Trend Test

The MK test was conducted based on two hypotheses. The null hypothesis (Ho) prompts that there is no trend in NDVI and EVI. The alternative hypothesis (H1) expresses that there is an increasing or decreasing trend in NDVI and EVI. Below is a list of the NDVI and EVI results from the MK test are shown in Table 2.

**Table 2:** Result of Mann-Kendall trend test.

Vegetation Index	Mann-Kendall Statistics	Kendall's Tau	Variance (S)	p-Value	Model Explanation	Presence of Trend
NDVI	150	0.7894737	950	0.000001337	Reject H_o_	Yes
EVI	144	0.7578947	950	0.000003492	Reject H_o_	Yes

The test was done at a 5% significance level. The table shows that both NDVI and EVI have a p-value less than 0.05, which rejects the null hypotheses (Ho). That means there are trends in both NDVI and EVI.

#### 3.5.2 Sen's slope calculation

Sen's slope was applied in this study to calculate the magnitude of the trend of NDVI and EVI. Sen's slope test results for NDVI and EVI are shown Table 3.

**Table 3:** Results of Sen's slope calculation.

Vegetation Index	Sen's Slope (Q_i_)	Sign of Q_i_	Types of Trends	Rate of Growth
NDVI	0.004241525	Positive (+)	Increasing	0.004241525/year
EVI	0.002566744	Positive (+)	Increasing	0.002566744/year

The value of Sen's slope for both NDVI and EVI is positive. That means there is an increasing trend in vegetation indexes between 2001 and 2020. The rate of growth for NDVI and EVI is 0.004241525/year and 0.002566744/year, respectively.

## 4 Discussion

Bangladesh has a temperate climate due to its geographical location. Between June and September, the most amount of rainfall occurs in Bangladesh. The peak evapotranspiration happens between June and October. Most of the time, the linear graph of evapotranspiration exceeds the bar plots of precipitation. Temperature, unlike precipitation and ET, showed the fewest fluctuation in the last two decades, though there were seasonal variations.

Using two separate vegetation indices, namely the NDVI and EVI, the greenness of the vegetation over Bangladesh was examined between 2001 and 2020. In the Mann-Kendall trend test, both NDVI and EVI have p-value less than 0.05, which rejects the null hypotheses (H_o_). That means there is a presence of trends in both NDVI and EVI. The value of Sen's slope for both NDVI and EVI is positive. This agrees with earlier research conducted on the Indian subcontinent [10]. The average NDVI and EVI increased from 0.47 to 0.53 and 0.289 to 0.31, respectively. The findings of earlier studies in comparable circumstances support this tendency [38]. From 2001 to 2020, the NDVI has regularly outclassed EVI in Bangladesh. This is also similar with a previous result [10].

There is a link between the EVI and hydroclimatic factors. The EVI has done brilliantly with ET, with a value of r^2^ = 0.373, as opposed to the NDVI, with a value of r^2^ = 0.17. Only 17% of the fluctuation in NDVI can be explained by evapotranspiration. There was no obvious correlation between the NDVI and precipitation. The findings of a prior study that was done in the same area ensure this conclusion [39]. In compared to the other two vegetation indices, the EVI seems to have a stronger association with ET and precipitation than the NDVI. The EVI index illustrated a strong correlation with temperature and precipitation, which is also consistent with another remote sensing-based research [40].

## 5 Conclusion

Ecological balance and environmental sustainability depend heavily on vegetation. For regional ecological management, it is vital to understand why and how vegetation evolves. Over the specified period, both vegetation indexes displayed a swelling tendency in plant greening. This implies that in the last two decades, Increases in EVI and NDVI have been seen in Bangladesh., and the NDVI has consistently exceeded the EVI. In both Sen's slope calculation and the Mann-Kendall trend test, the calculated values indicate the presence of a trend, and that trend is positive. The EVI seems to perform better with hydroclimatic factors, indicating that the EVI can perform better than the NDVI in change detection in vegetation. Both the NDVI and the EVI have displayed a one-to two-month lag with precipitation because there is no clear relation between vegetation greenness and precipitation. This study will be helpful for understanding long-term changes in vegetation in Bangladesh.

## Author contribution statement

Swadhin Das: Conceived and designed the experiments; Performed the experiments; Analyzed and interpreted the data; Contributed reagents, materials, analysis tools or data; Wrote the paper.

Showmitra Kumar Sarkar: Conceived and designed the experiments; Analyzed and interpreted the data; Wrote the paper.

## Data availability statement

Data will be made available on request.

## Declaration of competing interest

The authors declare that they have no known competing financial interests or personal relationships that could have appeared to influence the work reported in this paper.
